# Characterization of Polymorphic Microsatellites in the Giant Bulldog Ant, *Myrmecia brevinoda* and the Jumper Ant, *M. pilosula*

**DOI:** 10.1673/031.011.7101

**Published:** 2011-05-29

**Authors:** Zeng-Qiang Qian, F. Sara Ceccarelli, Melissa E. Carew, Helge Schlüns, Birgit C. Schlick-Steiner, Florian M. Steiner

**Affiliations:** ^1^ School of Marine and Tropical Biology, and Comparative Genomics Centre, James Cook University, Townsville, QLD 481 I, Australia; ^2^ Departamento de Zoología, Instituto de Biología, Universidad Nacional Autónoma de Mexico, C.P. 04510 México, D.F., México; ^3^ Centre for Environmental Stress & Adaptation Research, Bio21 Institute, The University of Melbourne, Melbourne, VIC 3010, Australia; ^4^ Molecular Ecology Group, Institute of Ecology, University of Innsbruck, 6020 Innsbruck, Austria

**Keywords:** codominant markers, colony structure, *Myrmecia pyriformis*, Myrmeciinae, *Nothomyrmecia macrops*

## Abstract

The ant genus *Myrmecia* Fabricius (Hymenoptera: Formicidae) is endemic to Australia and New Caledonia, and has retained many biological traits that are considered to be basal in the family Formicidae. Here, a set of 16 dinucleotide microsatellite loci were studied that are polymorphic in at least one of the two species of the genus: the giant bulldog ant, *M. brevinoda* Forel, and the jumper ant, *M. pilosula* Smith; 13 are novel loci and 3 are loci previously published for the genus *Nothomyrmecia* Clark (Hymenoptera: Formicidae). In *M. brevinoda*, the total of 12 polymorphic microsatellites yielded a total of 125 alleles, ranging from 3 to 18 with an average of 10.42 per locus; the observed and expected heterozygosities ranged from 0.4000 to 0.9000 and from 0.5413 to 0.9200, respectively. In *M. pilosula*, the 9 polymorphic loci yielded a total of 67 alleles, ranging from 3 to 12 with an average of 7.44 per locus; the observed and expected heterozygosities ranged from 0.5625 to 0.9375 and from 0.4863 to 0.8711, respectively. Five loci were polymorphic in both target species. In addition, 15 out of the 16 loci were successfully amplified in *M. pyriformis.* These informative microsatellite loci provide a powerful tool for investigating the population and colony genetic structure of *M. brevinoda* and *M. pilosula*, and may also be applicable to a range of congeners considering the relatively distant phylogenetic relatedness between *M. pilosula* and the other two species within the genus *Myrmecia.*

## Introduction

Studying ‘primitive’ ants has drawn wide attention from myrmecologists and other scientists because it may provide valuable insight into the origin and evolution of more derived social behaviors, life histories, and morphologies found in other ants (Haskins 1970). Ants of the genus *Myrmecia* Fabricius (Hymenoptera: Formicidae), colloquially known as bulldog and jumper ants, are, together with the monotypic genus *Nothomyrmecia* Clark, the only extant representatives of the formicid subfamily Myrmeciinae. The genus *Myrmecia* comprises 9 recognized species groups and 90 described species, all of which are endemic to Australia with just one of them present in New Caledonia (Ogata 1991; Hasegawa and Crozier 2006; Bolton et al. 2007). Although they were for a long time thought to be among the most basal formicids (Ogata 1991), this status has been rigorously rejected by recent phylogenetic studies (Ward and Brady 2003; Brady et al. 2006; Moreau et al. 2006; Rabeling et al. 2008; Moreau 2009). However, these aggressive ants have retained many biological traits that are considered to be ancestral in the family Formicidae. These include, for instance, the limited queen-worker divergence (Dietemann et al. 2004), the solitary foraging behavior using primarily visual and tactile cues (Haskins and Haskins 1950; Hölldobler and Wilson 1990), and its apparent inability to communicate stimuli among individuals (Haskins and Haskins 1950). From studies of the social organization of *Myrmecia* ants, one can hope to learn more about the early stages of formicid social evolution (Heinze 2008).

A great body of cytogenetic studies have been carried out on the genus *Myrmecia* (reviewed by Lorite and Palomeque 2010), but to date, the availability of molecular-genetic markers has been poor for this genus. Of 25 isozymes assayed by Craig and Crozier (1979), only one proved polymorphic and useful in *M. pilosula.* Due to their highly polymorphic and codominant nature, microsatellites have found wide applications in revealing the social structure of social insects (e.g. Wiernasz et al. 2004; Schlüns et al. 2009). Successful crosstaxa amplifications of 11 *Nothomyrmecia macrops* microsatellites in *M. forficata* were reported, but no details were made available regarding their polymorphisms, heterozygosity, linkage disequilibrium, and deviation from Hardy-Weinberg equilibrium (HWE) (Sanetra and Crozier 2000). Similar transferability was reported for 3 microsatellites, which were originally designed from the publicly available expressed sequence tags (ESTs) of *Solenopsis invicta* (Formicidae: Myrmicinae), but successfully amplified in *M. brevinoda* (Qian et al. 2009).

Here, a set of 16 dinucleotide microsatellite loci are presented that are polymorphic in the giant bulldog ant, *M. brevinoda* Forel (from the *M. gulosa* species group), and/or the jumper ant, *M. pilosula* Smith (from the *M. pilosula* species group), for the analysis of the population and colony genetic structure of these ants. The set consists of 10 novel loci isolated from an enriched genomic library of *M. brevinoda*, 3 novel loci isolated from a partial genomic library of *M. pyriformis* (from the *M. gulosa* species group), and 3 loci previously published for the genus *Nothomyrmecia.* All loci were also examined for their amplifiability in *M. pyriformis.*

## Materials and Methods

### Insect samples and DNA extraction

Samples of *M. brevinoda* were collected from a population in Paluma (Queensland, Australia; 19.0206° S/146.1433° E) in November 2009. Workers of *M. pilosula* were sampled from a population in Mongarlowe (New South Wales, Australia; 35.4647° S/149.9381° E) in February 2008. The sampling of *M. pyriformis* was conducted in the Wildlife Reserve of La Trobe University (Victoria, Australia; 37.7166° S/145.0533° E) in September 1999.

Genomic DNA of *M. brevinoda* and *M. pilosula* were prepared with Puregene DNA Isolation Kit (www.qiagen.com) following the manufacturer's protocol. DNA extraction of *M. pyriformis* was conducted using a standard phenol chloroform-based method which incorporated an RNase step (Sambrook et al. 1989).

### Microsatellite isolation

The isolation of microsatellite loci in *M. brevinoda* followed the standard protocol for microsatellite-enriched library construction (Bloor et al. 2001). Approximately 6 µg of genomic DNA was cut with the restriction enzyme *Rsa*I (New England BioLabs, www.neb.com). Adaptor-ligated DNA fragments ranging in size from 400 to 1000 bp were selected and enriched for (AG)_15_ repeats using M-280 Dynabeads (Invitrogen, www.invitrogen.com). The DNA was then ligated using the pGEM-T vector (Promega, www.promega.com) and transformed into JM109 *Escherichia coli* Competent Cells (Promega) for cloning. 352 clones were picked, of which 96 were selected for sequencing (from 175 shown to have an insert). Primers were then designed from 39 sequences using the program OLIGO v4.0 (Molecular Biology Insights, www.oligo.net). These candidate microsatellite loci were selected based on the availability of flanking regions suitable for primer design.

A partial genomic library was constructed for the isolation of microsatellite loci in *M. pyriformis.* Approximately 2 µg of purified genomic DNA was double digested with restriction enzymes *Hae*III and *Sau*3AI. Digests were run on a 1% agarose gel, and digest products ranging in size from 300 to 800 bp were excised from the gel. Size-selected digest products were then ligated into a *Bam*HI and *Hindi* digested pUC19 vector. Ligations were desalted and transformed into electrocompetent *E. coli* JM109 cells (Promega). *E. coli* colonies were grown on LB plates with ampicillin/IPTG/X-Gal media. Colonies were lifted onto N+ hybond membranes. Microsatellite probes (AC)_10_ and (AG)_10_ were end-labeled with [_C33_P] adenosine triphosphate (ATP) and hybridized to membranes overnight at 55° C. Membranes were exposed to a autoradiograph film, and the film was aligned back to colonies after exposure. Positive colonies were re-screened to confirm their status. Positive clones were sequenced using an *f*mol sequencing kit (Promega), and 12 primers were designed using OLIGO v4.0.

In addition, 14 previously published microsatellites, developed for *N. macrops* (Sanetra and Crozier 2000), were tested in this study to check their cross-taxa transferability and utility in the two target species. The forward primer of each locus was synthesized with a 5′-end 17-base tag to enable the labelling strategy of Shimizu et al. (2002) (Table 1).

### PCR amplification and genotyping analysis

In all, 65 microsatellite loci (39 isolated from *M. brevinoda*; 12 from *M. pyriformis*; 14 from *N. macrops*) were assayed against 20 individuals from 20 nests of *M. brevinoda* and 16 individuals from 16 nests of *M. pilosula.* The resultant polymorphic loci were also examined for their amplifiability in 2 individuals from a single nest of *M. pyriformis.* PCR amplifications were performed in the thermal cycler GeneAmp PCR System 9700 (PE Applied Biosystems, www.appliedbiosystems.com), using the following protocol: a final volume of 15 µl containing 1× PCR buffer, ∼50 ng of genomic DNA, 1.35 mM MgCl_2_, 0.2 mM each of dNTPs, 0.3 U Taq polymerase (Invitrogen), 0.4 µM universal fluorescent primer (GGTGGCGACTCCTGGAG, 5′ labeled with TET, HEX or FAM), 0.1 µM tagged primer, and 0.4 µM untagged primer. The PCR profile was as follows: initial denaturation at 94° C for 3 min; followed by 35 cycles of 94° C for 30 s, appropriate annealing temperature (see Table 1 for details) for 30 s, 72° C for 45 s, and last synthesis at 72° C for 7 min. Fluorolabeled PCR products were cleaned by centrifugation through 300 µl of Sephadex G-50 and multiplexed, and ET-400R size standard was added before genotyping on MegaBACE 1000 (Amersham Biosciences, www.gelifesciences.com).

**Table 1.**
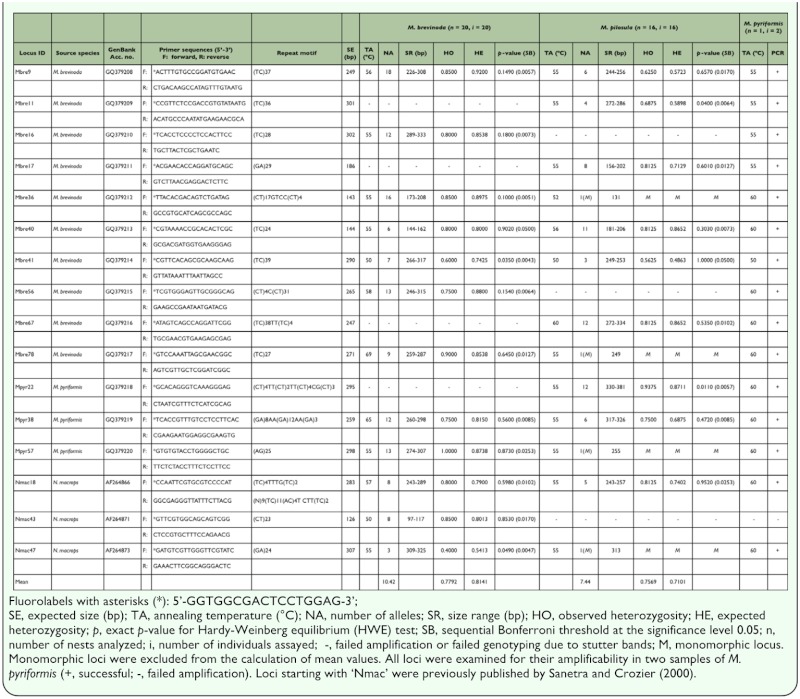
Characterization of polymorphic microsatellite loci in *Myrmecia brevinoda* and *M. pilosula*, and amplifiability in *M. pyriformis.*

**Table 2.**
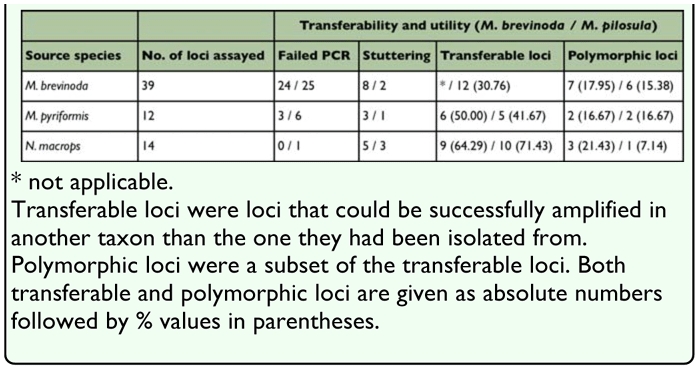
Transferability and utility of candidate microsatellite loci in *M. brevinoda* and *M. pilosula*

### Data analysis

The program PowerMarker v3.0 (Liu and Muse 2005) was employed to estimate various parameters: the number of alleles per locus (*N*A), observed (*H*O) and expected (*H*E) heterozygosities, deviation from Hardy-Weinberg equilibrium (HWE), and linkage disequilibrium. For the detection of linkage disequilibrium and the deviation from HWE, the sequential Bonferroni correction for multiple comparisons was applied at the significance level of 0.05 (Holm 1979). In addition, the possible presence of null alleles was checked using the program Micro-Checker v2.2.3 (Van Oosterhout et al. 2004).

## Results

Forty-nine loci were discarded due to poor amplification, severe stuttering or monomorphism (at the 95% criterion). Sixteen loci (10 isolated from *M. brevinoda*; 3 from *M. pyrifomis*; 3 from *N. macrops*) yielded clear and interpretable peaks and were polymorphic in at least one of the two target species, *M. brevinoda* and *M. pilosula* (Table 1; Table 2). Of these loci, 12 and 9 generated polymorphic products in *M. brevinoda* and *M. pilosula*, respectively, with 5 of them proving useful in both species. Notably, 64.29% (9/14) and 71.43% (10/14) of the candidate loci originally isolated from *N. macrops* were successfully amplified in *M. brevinoda* and *M. pilosula,* respectively (Table 2). In addition, 15 out of the 16 loci (93.75%) proved amplifiable in *M. pyriformis* (Table 1), the failed locus being one developed for *N. macrops.*

In *M. brevinoda*, the 12 polymorphic microsatellites yielded a total of 125 alleles, ranging from 3 (Nmac47) to 18 (Mbre9) with an average of 10.42 per locus; the observed and expected heterozygosities ranged from 0.4000 to 0.9000 and from 0.5413 to 0.9200, with an average of 0.7792 and 0.8141, respectively. In *M. pilosula*, the 9 polymorphic loci generated a total of 67 alleles, ranging from 3 (Mbre41) to 12 (Mbre67/Mpyr22) with an average of 7.44 per locus; the observed and expected heterozygosities ranged from 0.5625 to 0.9375 and from 0.4863 to 0.8711, with an average of 0.7569 and 0.7101, respectively. Following the sequential Bonferroni correction, no significant deviation from HWE or linkage disequilibrium was observed in any of the 16 loci in any of the species. Possible occurrence of null alleles was suggested in one locus (Nmac47) for *M. brevinoda*, but not in any locus for *M. pilosula.*

## Discussion

In this study, a set of polymorphic dinucleotide microsatellite loci suitable for population genetic studies of *Myrmecia brevinoda* and *M. pilosula* were collated. The levels of heterozygosity reported here were comparable to those previously reported for the consubfamilial dinosaur ant, *Nothomyrmecia macrops* (Myrmeciinae) (Sanetra and Crozier 2000), and one species of the subfamily Ponerinae s.str. (Bolton 2003), which diverged early from other ants (Moreau 2009), i.e., *Diacamma cyaneiventre* (Ponerinae) (Doums 1999). The heterozygosity levels found were higher, however, than those for the only other ant from Ponerinae for which microsatellite data are available, i.e., *Diacamma ceylonense* (Gopinath et al. 2001).

The success rates in screening the candidate microsatellites in *M. brevinoda* and *M. pilosula* were rather low. Interestingly, those loci originally isolated from *N. macrops* displayed a high level of cross-generic transferability, which was substantially higher than that reported in the cross-subfamilial transferability of EST-derived microsatellites from *Solenopsis invicta* (Myrmicinae) to *M. brevinoda* (25.00%, 3/12) (Qian et al. 2009). Likely, this reflects the much closer phylogenetic relatedness among the genera of the same subfamily as compared to that of genera from different subfamilies. However, out of the 10 transferable loci, only one proved polymorphic in *M. pilosula*, in line with the widely held viewpoint that the allelic diversity of transferable microsatellites is closely correlated with the phylogenetic relatedness between the donor and acceptor species (Decroocq et al. 2003). In addition, 15 out of the 16 loci were successfully amplified in *M. pyriformis*, suggesting the potential applicability in this species. Considering the usefulness of the loci in two different *Myrmecia* species groups (*M. gulosa* species group: *M. brevinoda, M. pyriformis; M. pilosula* species group: *M. pilosula*), which are relatively distant phylogenetically (Hasegawa and Crozier 2006), the loci may also be applicable to other species groups of the genus.

It should be noted that constant PCR conditions were used to evaluate the cross-taxa transferability and utility of these microsatellites in the target species. Further optimization of the PCR protocol might potentially increase the success rate of marker development. These polymorphic loci will be employed to investigate the breeding system, population, and colony genetic structure in *M. brevinoda* and *M. pilosula.*

## Abbreviations

**EST**,: expressed sequence tag;
**HWE**,: Hardy-Weinberg equilibrium
